# A Small U-Shaped Bending-Induced Interference Optical Fiber Sensor for the Measurement of Glucose Solutions

**DOI:** 10.3390/s16091460

**Published:** 2016-09-09

**Authors:** Yu-Lin Fang, Chen-Tung Wang, Chia-Chin Chiang

**Affiliations:** Department of Mechanical Engineering, National Kaohsiung University of Applied Sciences, Kaohsiung 807, Taiwan; fangiack@gmail.com (Y.-L.F.); 1101403104@gm.kuas.edu.tw (C.-T.W.)

**Keywords:** whispering gallery mode, optical fiber sensor, glucose

## Abstract

The study proposes a small U-shaped bending-induced interference optical fiber sensor; this novel sensor is a probe-type sensor manufactured using a mechanical device, a heat source, optical fiber and a packaging module. This probe-type sensor overcomes the shortcomings of conventional optical fibers, including being difficult to repair and a tendency to be influenced by external forces. We manufactured three types of sensors with different curvature radiuses. Specifically, sensors with three radiuses (1.5 mm, 2.0 mm, and 3.0 mm) were used to measure common water and glucose solutions with concentrations of between 6% and 30% (the interval between concentrations was 4%). The results show that the maximal sensitivity was 0.85 dB/% and that the linearly-dependent coefficient was 0.925. The results further show that not only can the small U-shaped bending-induced interference optical fiber sensor achieve high sensitivity in the measurement of glucose solutions, but that it can also achieve great stability and repeatability.

## 1. Introduction

Optical fibers have often been utilized in communications [1] and data transmission [2]; however, optical fiber technology has advanced substantially in recent years, increasing its value and expanding its potential uses even further. Optical fiber has various advantages. For example, it is light, small in size, highly sensitive, highly flexible [3], highly resistant to electromagnetic interference (EMI) [4], and can be embedded into or attached to a wide range of structures [5]. Thus, optical fiber has strong potential for further development and application. In recent years, for example, various sensors manufactured from optical fiber have been used to not only measure various physical properties (such as temperature change [6], strain [7], vibration effects [8], etc.), but also concentration changes of various liquids [9].

Rayleigh [10] (an English physicist) proposed the whispering gallery mode (WGM) theory, and his research investigated, among other things, the transmission phenomena of acoustic waves along a curved wall. Subsequently, numerous scholars have investigated how to transmit acoustic waves in bent optical fiber according to Rayleigh’s theory. In 2010, Wang et al. [11] proposed using a WGM refractive index sensor to measure the refractive indices of external media. In a related experiment, they used three optical fibers with different curvatures to measure the refractive indices of seven solutions with different concentrations at room temperature. In manufacturing each sensor, they removed the protective layer of the optical fiber and bent the optical fiber to form a ring with a diameter of 19 mm, and then used glue to ensure that the shape of the optical fiber would be maintained. Next, the finished sensor was placed in the experimental solution for measurement. The experimental results showed that the refractive index sensitivity of the optical fiber sensor with a diameter of 19.3 mm could reach as high as 725.76 nm/RIU.

In 2012, Mathew et al. [12] proposed the use of bent optical fiber to manufacture a humidity sensor for use in a humidity measurement system. They packaged an optical fiber in a sensing layer and then placed the fiber on a movable platform in order to change the radius of the optical fiber through the movements of the platform. Next, they prepared several measurement environments with different humidity in order to observe the rate of energy change in the fiber; the advantages of the system included its low cost and quick rate of response, which made it very suitable as a humidity measurement sensor. In 2013, Nishimura and Tanabe [13] proposed the use of a spherical chamber made of optical fiber to measure the differences between tap water and pure water. More specifically, they changed the refractive index of the WGM and the surface of the chamber to attract nanoparticles in order to measure samples of tap water and pure water. The results showed that the device exhibited a wavelength shift of 89 pm when measuring water, and the authors thus suggested that it had potential for use in monitoring water purity.

In 2013, Kwon and Steier [14] proposed a micro-ring-resonator-based glucose sensor consisting of two different micro-ring resonators. The authors measured both the temperature and concentration of glucose solutions simultaneously. In 2014, Bhardwaj et al. [15] proposed the dual taper-like fiber probe glucose sensor based on a Mach–Zehnder Interferometer (MZI) using the arc fusion splicer. Relatively high sensitivities are achieved by using the intensity interrogation methods. The sensitivity of the device is about 376.12 nm/RIU.

In 2014, Chao [16] proposed a U-shaped bending optical fiber glucose concentration sensor. The results showed that as the tested glucose concentration increased from 0% to 50%, the spectrum shifted from short to long wavelengths. When the fiber diameter was 64 μm and bend radius was 3.5 mm, the optimal glucose concentration sensitivity was 0.520 nm/%.

The results of the studies reviewed above indicate that bending optical fiber sensors could measure changes in the refractive index of solutions. In fact, researchers have already found that sensors utilizing this design exhibit good sensitivity in solution measurements, including measurements of solution concentration and water quality. The present study expanded upon such research by using small U-shaped bending-induced interference optical fiber sensors of different sizes to test glucose solutions with different concentrations.

## 2. Materials and Sensor Manufacturing Process 

**Figure 1:**
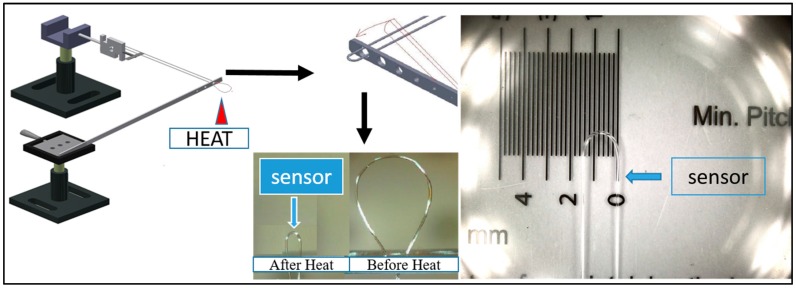
Schematic view of manufacturing process of small U-shaped bending-induced interference glucose concentration sensor.

As shown in Figure 1, we removed 60 mm of optical fiber protective layer from the middle section of a 300 mm-long single mode fiber. Next, we fixed the two ends of the fiber to a movable platform consisting in part of a 300 mm-long flexible aluminum board. More specifically, we fixed the ends of the fiber to a tension meter, and set the diameter of the optical fiber so that it would be 7 mm when the tension meter reached 12 N. Next, we heated up the beat ends of the optical fiber. During the manufacturing process, the aluminum board bounced off because of the release of the potential energy caused by the tension, which in turn caused the optical fiber to pass through the holes and assume the diameter and the size that we had designed. Finally, the desired probe-type U-shaped fiber component was finished.

We used quartz glass to package the finished probe-type U-shaped fiber component, maintained the distance required to remove the optical fiber at the outlets of the capillary, and then used transparent super glue to fix the outlets of the tube in order to form an independent sensor; the optical fiber was fixed according to the design specifications. The advantage of this design was in the further fixation of the probe head, which allowed the influence of human-made and environmental factors to be reduced. At the same time, the optical fiber would not crack when used to measure a given liquid.

## 3. Experiment

**Figure 2:**
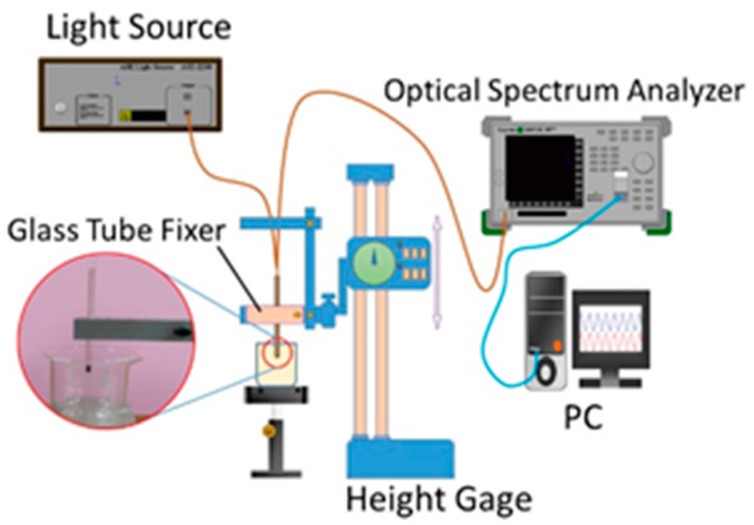
Experimental set-up for the small U-shaped bending-induced interference optical fiber sensor glucose solution concentration refractive index experiment.

In the experiment in which the small U-shaped bending-induced interference optical fiber sensor was used to measure the refractive indices of glucose solutions, the sensor was first immersed into liquids with different refractive indices. The relationship between the given refractive index and the wavelength was then determined according to the phase change during reflection due to the refractive index difference between the casing of the optical fiber and the external liquid. Figure 2 shows the set-up of the equipment that we used with the small U-shaped bending-induced interference optical fiber sensor in the glucose solution concentration refractive index experiment. First, we affixed the glass tube to the height gage with double circular bars. Next, we affixed the small U-shaped bending-induced interference glucose concentration sensor to the glass tube fixer; one end of the sensor was connected to the optical spectrum analyzer (OSA), and the other end of the sensor was connected to the light source. Next, the OSA transferred the signal into the spectrum signal, and then the spectrum signal was shown on the PC monitor. 

Regarding the glucose solutions that were measured, we prepared several glucose solutions with concentrations ranging from 6%–30% (with intervals of 4% between solutions). Each solution was then placed in turn on the height-adjustable measurement table, and then the distance between the glass tube fixer of the height gage and the fluid level of the glucose solution in the beaker was adjusted as necessary until the sensor was immersed in the solution, allowing the refractive index measurement of that solution to be conducted.

## 4. Results and Discussion

### 4.1. Concentration Refractive Index Measurement of Solutions to be Measured

The experimental process can be divided into two parts, including the glucose refractive index measurement by the Abbe refractometer and the water/glucose solution measurement by the small U-shaped bending-induced interference concentration sensor (the diameter and the bent radius of the optical fiber used were 125 µm and 1.5 mm, respectively).

**Figure 3:**
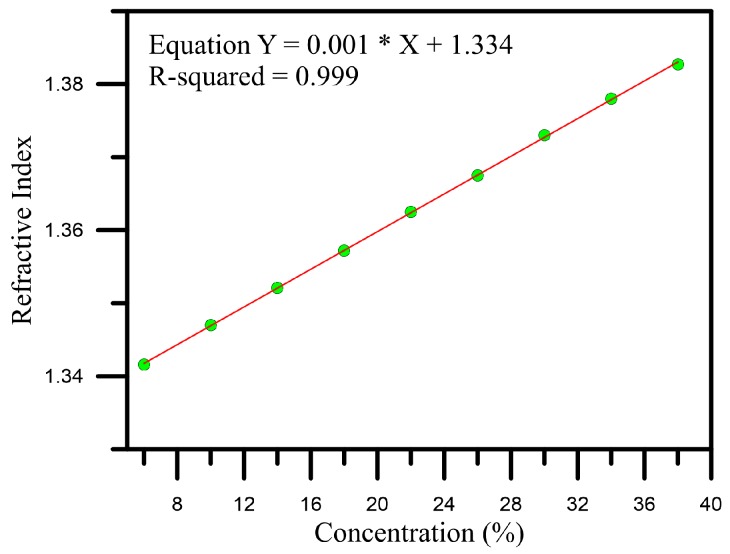
Diagram of the relationship between glucose solution concentration and refractive index.

First, we use the Abbe refractometer to measure the liquid refractive index of each glucose solution, and the measurement results indicated a linear relationship between the concentration and the refractive index, as shown in Figure 3. In addition, the sensitivity was 0.001 dB/%, and the linearly-dependent coefficient R^2^ was 0.999, showing that the solutions used in the experiment can be recognized according to the solution accuracy.

### 4.2. Concentration Test of Water/Glucose Solution

**Figure 4:**
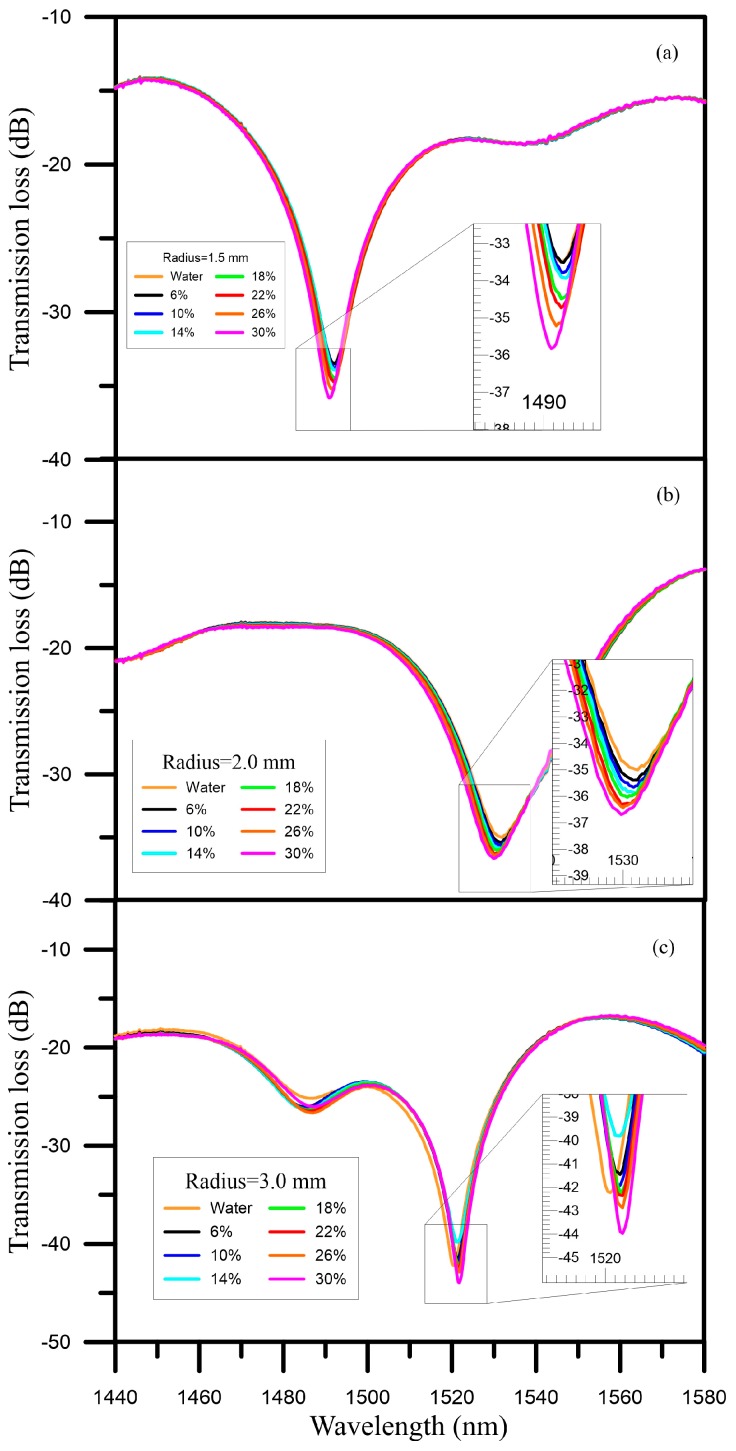
Concentration sensing spectrum diagram of glucose solution for (**a**) a bent radius D = 1.5 mm; (**b**) a bent radius D = 2.0 mm; and (**c**) a bent radius D = 3.0 mm.

We first immersed the sensor with a bent radius of 1.5 mm into water, and found that the central wavelength was 1491.75 nm and the transmission loss was −33.075 dB. Next, we measured glucose solutions with concentrations of 6%–30%. When the concentration of the glucose solution was 6%, the central wavelength was 1492.0 nm. In contrast, when the concentration of the glucose solution was 30%, the central wavelength was 1492.0 nm, but the wavelength shift was 1.75 nm. Thus, we could see that the long wavelength was shifted toward the direction of the short wavelength at the concentration wavelength position. In terms of the transmission loss, as the concentration was raised from 6% to 30%, the transmission loss ranged from −33.509 dB to −35.827 dB; thus, the energy loss tended to increase, as shown in Figure 4a. 

When we used the sensor with a bent radius of 2.0 to measure the solutions, the wavelength also tended to be shifted toward the short wavelength as the concentration increased; specifically, the wavelength was shifted from 1531.25 nm (6%) to 1529.75 nm (30%). As such, the wavelength shift was 1.5 nm, and the transmission loss grew from 35.395 dB to 36.687 dB, as shown in Figure 4b.

When we used the sensor with bent radius of 3.0 mm to measure the solutions, the wavelength was shifted toward the long wavelength as the concentration was increased. Specifically, the wavelength was increased from 1521.5 nm (6%) to 1521.75 nm (30%). The wavelength shift was 0.25 nm, and the transmission loss decreased from −41.453 dB to −41.275 dB, as shown in Figure 4c. However, when the concentration of the solution was 14%, the wavelength was 1521.5 nm and the transmission loss was −39.808 dB.

**Figure 5:**
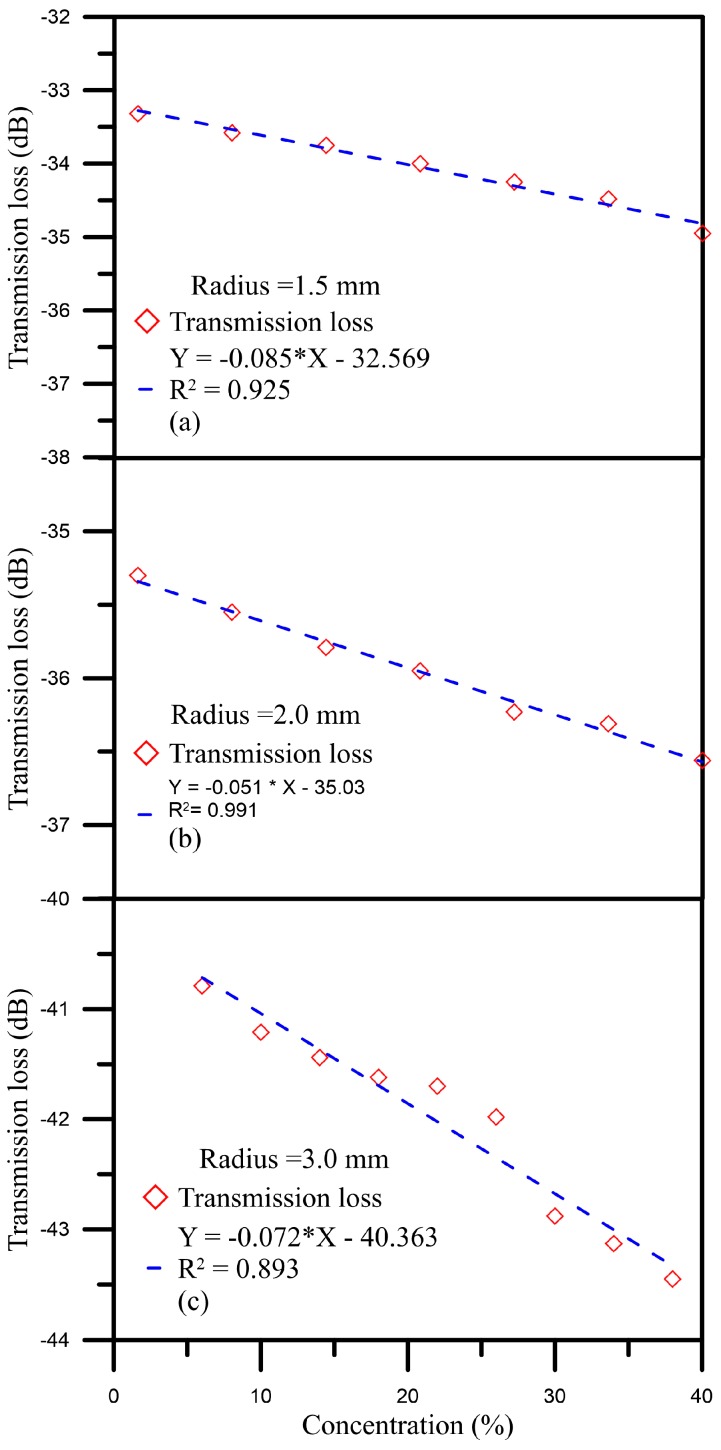
Diagram of the relationship between glucose solution concentration and transmission loss.

When we used the sensor with a bent radius of 1.5 mm to measure the glucose solutions with concentrations between 6% and 30%, the energy transmission loss was −33.509 dB. As the concentration was increased, a maximal energy loss of −35.827 dB was found when the concentration of the glucose solution was 30%, and the energy loss reached up to 2.291 dB, as shown in Figure 5a. Furthermore, the sensitivity was 0.085 dB/% and the linearity was 0.925. The results for the sensor with a bent radius of 2.0 mm are shown in Figure 5b; the energy loss was 1.26 dB, the sensitivity was 0.051 dB/%, and the linearity was 0.991. For the sensor with a bent radius of 3.0 mm, the transmission loss was 2.24 dB, the sensitivity was 0.081 dB/%, and the linearity was 0.947, as shown in Figure 5c. According to the experimental results above, these sensors are very sensitive to changes in concentration.

**Figure 6:**
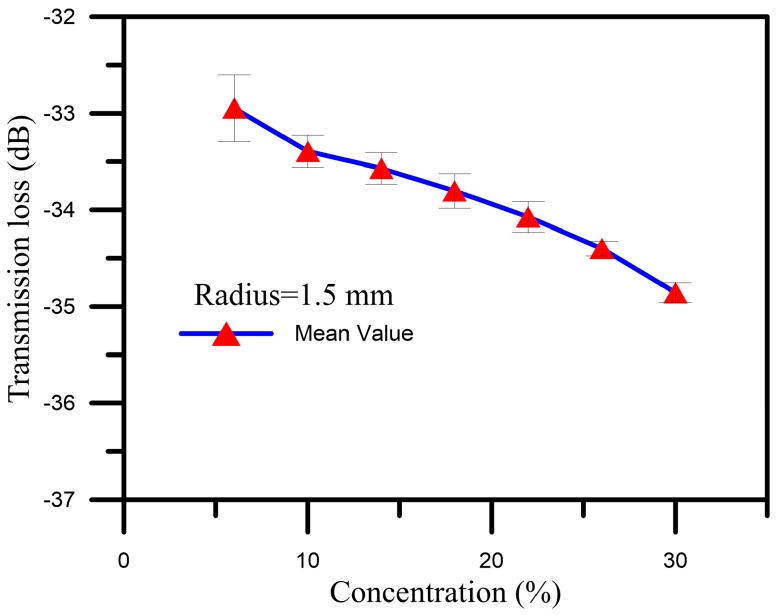
Diagram of the standard deviation analysis results for the obtained data.

Next, we conducted repeatability and reproducibility experiments for the sensors. We used a sensor with a bent radius of 1.5 mm to measure glucose solutions with concentrations of 6%–30% for three cycles, and then performed a standard deviation analysis of the obtained data, the results of which are shown in Figure 6. This analysis found that the standard deviation of the transmission loss was within one standard deviation, as shown in Table 1. Accordingly, we can conclude that the sensors with radiuses of 1.5 mm, 2.0 mm, and 3.0 mm have good reproducibility, and that these sensors can be repeatedly used for measurement.

## 5. Conclusions

This study utilized small U-shaped bending-induced interference optical fiber sensors. The respective bent radiuses of the sensors used were 1.5 mm, 2.0 mm, and 3.0 mm. Furthermore, the sensors were packaged in quartz glass tubes. We found that sensors manufactured in this way have greater storability, and that they do not suffer data distortion due to external forces; furthermore, such sensors can be manufactured in a short time and at a low cost.

In the experiments, we used glucose solutions to test the sensors. The measurement results showed that when the concentration of the glucose solutions was increased and when two sensors with bent radiuses of 1.5 mm were used to the measure the solution concentrations, the wavelength tended to be shifted from a long wavelength to a short wavelength. In terms of transmission loss, the result showed that the concentration wavelength and the transmission loss of the sensor with a bent radius of 1.5 mm was predictable. Moreover, the transmission loss standard deviation results showed that the accuracy of the sensor was high.

The study found that when used to measure glucose solution concentrations, the small U-shaped bending-induced interference optical fiber sensor with D = 1.5 mm achieved the best performance in terms of transmission loss and measurement accuracy. The above experiments further showed that the small U-shaped bending-induced interference sensor constitutes a highly-sensitive and convenient glucose concentration sensor. In addition, the sensor also exhibited good repeatability and stability. In summary, this type of sensor is an excellent sensor and can be further developed for use as a real-time concentration monitoring sensor.

## Figures and Tables

**Table 1:** Wavelength and transmission loss standard deviation table for measurements made using the sensor with a radius of 1.5 mm to measure glucose solutions with concentrations of 6%–30% for three cycles.

Concentration	6%	10%	14%	18%	22%	26%	30%
Transmission loss standard deviation (dB)	0.34	0.17	0.16	0.18	0.16	0.08	0.10
